# Effectiveness of the modified Seldinger technique for peripheral central catheter in newborns: a randomized clinical trial

**DOI:** 10.1590/0034-7167-2024-0189

**Published:** 2024-12-16

**Authors:** Izabela Linha Secco, Mitzy Tannia Reichembach Danski, Luana Lenzi, Higor Pacheco Pereira, Juliana Szreider de Azevedo, Letícia Pontes, Regiane Queiroz Afonso, Camila Fernanda da Silva Milani

**Affiliations:** I Hospital Infantil Waldemar Monastier. Campo Largo, Paraná, Brazil; II Universidade Federal do Paraná. Curitiba, Paraná, Brazil

**Keywords:** Comparative Effectiveness Research, Peripheral Catheterization, Newborn, Technology, Randomized Controlled Trial., Efectividad, Cateterismo Periférico, Recién Nacido, Tecnología, Ensayo Clínico Controlado Aleatorio.

## Abstract

**Objectives:**

to evaluate the effectiveness of peripheral central catheterization by comparing the modified Seldinger technique and the conventional technique in critically ill newborns.

**Methods:**

randomized unmasked clinical trial conducted in a public children’s hospital. Participation of 111 newborns with randomized allocation, 56 in the control group (conventional technique) and 55 in the experimental group (modified Seldinger). Success and absence of complications were evaluated as primary outcomes. The pain scale, difficulty in hemostasis, procedure time and number of punctures were considered secondary outcomes.

**Results:**

there was no statistical significance between groups, either for success (p=0.705) or absence of complications (p=0.347). A lower pain score, improved hemostasis, increased assertiveness with fewer punctures and reduced procedure time were not observed in the experimental group.

**Conclusions:**

the modified Seldinger technique did not prove to be a more effective insertion technology compared to the conventional method. Brazilian Clinical Trial Registry: RBR-69vks36.

## RESUMEN

**Objetivos:**

evaluar la efectividad de la cateterización central periférica entre la técnica Seldinger modificada y la técnica convencional en recién nacidos gravemente enfermos.

**Métodos:**

ensayo clínico aleatorio, abierto realizado en un hospital público de niños. Participaron 111 recién nacidos con asignación aleatoria, 56 en el grupo control (técnica convencional) y 55 en el grupo experimental (Seldinger modificada). El éxito y la ausencia de complicaciones se evaluaron como resultados primarios. La escala de dolor, la dificultad en la hemostasia, el tiempo del procedimiento y el número de punciones se consideraron resultados secundarios.

**Resultados:**

no hubo significación estadística entre los grupos, ni para el éxito (p=0,705) ni para la ausencia de complicaciones (p=0,347). No se observó menor puntuación de dolor, mejoría de la hemostasia, mayor asertividad con menos punciones y una reducción del tiempo del procedimiento en el grupo experimental.

**Conclusiones:**

la técnica Seldinger modificada no demostró ser una tecnología de inserción más eficaz en comparación con el método convencional. Registro Brasileño de Ensayos Clínicos: RBR-69vks36.

## INTRODUCTION

Newborns (NBs) admitted to Neonatal Intensive Care Units (NICUs) require prolonged and safe intravenous therapy due to the predominance of medications and fluids that require long-term central administration^(1)^. Therefore, central line placement in NBs is essential.

Experts have advocated the early transition from infusion therapy to the Peripherally Inserted Central Catheter (PICC)^(2)^. The attractive features of PICC lines include bedside insertion, lower complication rate compared to other central devices, reduction of multiple punctures, discomfort and stress, in addition to preservation of the venous network for future use^(3)^.

Although this has become a feasible device, its use is not free of complications and challenges, both justified by the vulnerability of NB, especially premature infants. To begin with, the venous system is much more restricted in this population compared to other age groups^(4)^. Other attributes that make PICC insertion technically demanding in NB include: loose skin and subcutaneous tissue, smaller and barely visible blood vessels, lack of cooperation during the procedure, and different anatomy/physiology^(5)^.

These challenges have resulted in a failure rate of approximately 50% in catheterization on the first attempt^(6)^. The number of attempts is a known risk factor for mechanical, infectious, and painful complications, which can occur with each additional attempt during catheterization in neonatal patients. Therefore, success on the first attempt in all stages of the procedure is important. Although the overall incidence of successful catheterization is an important outcome, success in the first puncture is considered a more significant parameter in NB^(7)^.

The effectiveness of PICC insertion on the first attempt and the reduction of adverse events can be achieved through innovative technologies and practices, which have evolved considerably, all designed to improve intraprocedural steps^(6)^.

A technique known as Seldinger that exists since the 1950s revolutionized access to the venous network in critically ill patients, when a radiologist had the idea of using a guidewire after the needle puncture to guide the catheter into the blood vessel. With technological refinements, the technique has evolved significantly, resulting in less invasiveness in the insertion of central lines. After these improvements, the Seldinger technique was modified (Modified Seldinger Technique - MST) to also serve patients who require special care, such as NB^(8)^.

In contrast to the conventional technique, MST (microintroduction) brings numerous benefits, such as the insertion of a larger catheter caliber, reduced pain, bleeding and the risk of bloodstream infection^(9,10)^. And, most importantly, increased success in the first puncture, considering that the progression of the guidewire into the vessel increases the chance of the PICC being guided to the cavoatrial junction (CAJ)^(11)^. Assertiveness can still be maximized with the use of ultrasound, but the MST is independent of this equipment to be performed.

Although both insertion technologies coexist in NICUs, the conventional one is still the most prevalent. The only device in Brazil that meets the requirements for microintroduction for neonatology was launched in 2017 - the Per-Q-Cath^®^NeoKit PICC MST. Regarding the incorporation of new technologies for NB, having a technique that provides successful venous catheterization and reduces the chances of complications is of the utmost importance in the care of this population.

In view of the above, the study is justified for nursing practice because it includes a very fragile and vulnerable age group with peculiar characteristics not observed in any other patient population, especially the venous network. Furthermore, innovative practices in this clientele are initially used in older age groups, delaying their application in neonatal clinical practice.

## OBJECTIVES

To evaluate the effectiveness of peripheral central catheterization by comparing the MST and the conventional technique in critically ill NB through the success of PICC insertion and the occurrence of immediate and late complications related to these technologies.

## METHODS

### Ethical aspects

The study was conducted in accordance with national and international ethics guidelines, and was approved by the Research Ethics Committee of the Health Sciences Sector - Universidade Federal do Paraná (opinion is attached to this submission). The clinical trial was approved by the Brazilian Clinical Trials Registry (RBR-69vks36).

### Study design, period and location

This is an unmasked randomized clinical trial (RCT) conducted between June 2022 and November 2023 at a public children’s hospital that is a reference in the state of Paraná. The Consolidated Standards of Reporting Trials (CONSORT) was the guiding instrument for the study design.

### Population or sample, inclusion and exclusion criteria

The sample size was calculated based on a study developed in the United Kingdom^(12)^, where the incidence of successful catheterization on the first attempt was 72% with the MST versus 40% with the conventional technique. Using a significance level (α) of 5%, a statistical power of 90% (1-β), and considering a possible dropout rate of 5%, the required sample size was 57 participants for each group. However, based on the prediction that two experimental catheter kits would be expired when insertions began, the sample totaled 112 patients.

Patients admitted to the NICU from the first day of life onwards, who presented an indication for PICC according to the Michigan Appropriateness Guide for Intravenous Catheters in *pediatrics* (miniMAGIC) were included^(13)^. Written informed consent was obtained from all legal guardians of the patients. Patients with venous anomalies, skin infection, thrombocytopenia (≤ 50,000 mm^3^) and requiring double-lumen central venous catheterization were excluded.

### Study protocol

The randomization process was performed by an external researcher with experience in RCTs, using computer-generated software (RANDOM.ORG). Using opaque and tamper-proof envelopes with external numbers from 1 to 112, the group allocations were concealed with the following information: conventional technique (CG - Control Group) and MST (EG - Experimental Group).

Given that nurses handle the catheter kit before starting the procedure because this step is part of the pre-insertion protocol, they could not be blinded. Therefore, they could differentiate the techniques, since the MST kit has some items that are not present in the conventional kit.

For each PICC indication, the patient was subjected to the eligibility criteria. If eligible, the nursing assistant contacted the research team in advance. The team took the sealed brown envelope to the NICU, following the previously performed randomization, which determined in which group the patient in question would be included to later identify the insertion technology. A randomization diary with the number of patients and the group to which they belonged was created to assist in this control. It was filled out immediately after the research team opened the envelope.

Once randomly allocated to use one of the insertion techniques, two nursing assistants carefully evaluated the venous network and estimated catheter measurement. Next, they organized the materials and dressed in a maximum sterile barrier. At this time, the research team opened the envelope and demonstrated which kit should be made available (MST or conventional). At the same time, sedation and analgesia were prescribed, prepared and administered prior to insertion

The EG received the intervention using the MST technique, while the CG received the usual treatment, that is, the PICC insertion technique already in place at the study site. Using the conventional technique, the intervention occurred through peripheral venipuncture, one centimeter behind the desired insertion point. In the presence of blood reflux in the chamber of the needle introducer, the needle was separated from the peel-away dilator and the catheter was slowly inserted to the desired length. After the device had fully advanced, the peel-away was split until it was separated from the PICC. The catheter was stabilized by the fixation wings with sterile adhesive tape and compression at the insertion site was applied with sterile cotton to contain the bleeding. Finally, the sterile transparent cover was placed over the insertion bed and the location of the catheter tip was immediately requested and assessed by radiography before starting the intravenous infusion.

The intervention performed through the MST included peripheral venipuncture with a needle device independent of the peel-away dilator. With venous return, the guidewire was inserted through the needle until five centimeters remained externally. Once this progression was achieved without difficulty, the needle was withdrawn over the guidewire, which remained in the patient’s vessel to guide the placement of the peel-away dilator to the end of its length (over the guidewire). To insert the dilator, the nurse stretched the NB’s skin downwards to facilitate penetration into the skin. The guidewire was then removed and the catheter inserted to the desired length. The remaining steps were the same as those described for the conventional technique.

When notified by the nursing assistant, the research team began filling out the data collection instrument using information already described in the electronic medical record, such as patient characteristics and reasons for the PICC indication. Other variables of interest were recorded at the bedside by the team, during and after the procedure, including catheterization data and outcomes. If the procedure was successful, the patient was daily monitored by the researchers until the catheter was removed.

### Outcomes

The variables “insertion success” and “absence of immediate and late complications” were considered as primary outcomes. The first included the positioning of the PICC in CAJ and was assessed by a nurse or physician not participating in the study with the use of radiography immediately after the end of the procedure.

Regarding complications, the occurrence of hematoma, difficult-to-control bleeding and inadvertent arterial puncture were considered immediate complications, measured during bedside catheterization by the research team. Central line-associated bloodstream infection (CLABSI) was chosen as a late complication, as it is intrinsically related to the number of puncture attempts. This was determined through daily observation of the patient during the first two weeks of the PICC in situ, through information obtained from the electronic medical record and direct communication with the team of physicians and care nurse practitioners in the NICU.

Secondary outcomes were the pain scale during the procedure, difficulty in achieving hemostasis at the insertion site up to 48 hours, procedure time, and number of puncture attempts, all of which were also measured by the researchers. The Premature Infant Pain Profile (PIPP) scale was used to determine the presence of pain and grade it. The dressing was viewed on the first and second day after the procedure to determine whether or not local compression was needed to control bleeding. In cases of successful catheterization, the time in minutes was recorded from the first puncture attempt until the peel-away broke. In cases of unsuccessful catheterization, the total time was determined from the first to the last puncture attempt. The number of attempts was defined by the number of times the NB was punctured.

### Analysis of results and statistics

All variables of interest were transcribed and subsequently coded in a Microsoft Excel Office 365^®^ spreadsheet. The IBM SPSS Statistics version 29.0 was used in the statistical analysis of data.

The Kolmogorov-Smirnov test was used to assess the distribution pattern of continuous variables. The Kruskal-Wallis test was used in comparisons between nonparametric continuous variables, and the Pearson’s chi-square test in comparisons between categorical variables, which were described using absolute and relative frequency. Median and 95% confidence interval were used to describe continuous variables. The comparison of cumulative assertiveness and the number of attempts between the control and experimental groups was performed using the Kaplan-Meyer method.

A significance level of 5% (p<0.05) was considered in all analyses, reflecting a 95% confidence interval.

## RESULTS

**Figure 1:**
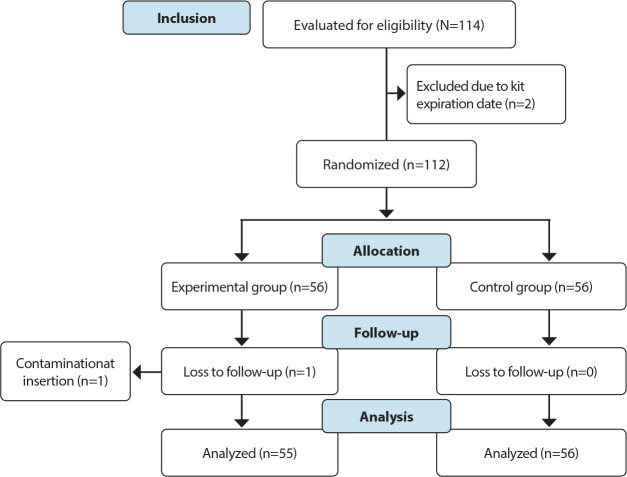
Flowchart of recruitment and allocation of study participants, Curitiba, Paraná, Brazil, 2023

Recruitment took place from June 2022 to November 2023; 56 NB were randomly assigned to the EG and 56 to the CG. As one catheter got contaminated during one of the insertions using the MST, a follow-up in the EG was lost (Figure 1).

Demographic data and clinical characteristics of each group are presented in Table 1. The NB did not differ significantly in terms of age, weight, mortality risk, medical diagnosis that culminated in PICC insertion, difficult venous access score, and reasons that indicated the need for percutaneous catheterization.

**Table 1:** Sociodemographic and clinical characteristics of newborns undergoing percutaneous catheterization, Curitiba, Paraná, Brazil, 2023

Variable	Control Group (n=56)		Experimental Group (n=55)		p^*^
	n (%)	Median (95%CI)	n (%)	Median (95%CI)	
Gestational age at birth (weeks)		36 (34-38)		36 (33-38)	0.785^1^
Age at insertion (days)		36 (23-43)		29 (11-59)	0.797^1^
Birth weight (grams)		2268 (1650-2780)		2355 (1420-2755)	0.969^1^
SNAPPE II scale^**^		8 (5-15)		5 (5-15)	0.255^1^
DIVA score^***^ ≥ 4	34 (48.6)		36 (51.4)		0.605^2^
Catheter indication					0.773^2^
Therapy incompatible with peripheral route	48 (49.0)		50 (51.0)		
Therapy ≥ 7 days	10 (47.6)		11 (52.4)		
Difficult venous access	37 (48.7)		39 (51.3)		
Diagnosis that culminated in catheter insertion					0.497^2^
Infectious and parasitic diseases	16 (48.5)		17 (51.5)		
Disorders of other endocrine glands	1 (100.0)		0 (0.0)		
Diseases of the nervous system	1 (33.3)		2 (66.7)		
Diseases of the respiratory system	5 (50.0)		5 (50.0)		
Diseases of the digestive system	3 (37.5)		5 (62.5)		
Diseases of the genitourinary system	2 (66.7)		1 (33.3)		
Conditions originating in the neonatal period	24 (60.0)		16 (40.0)		
Congenital malformations, deformities and chromosomal anomalies	4 (28.6)		10 (71.4)		

n - numberof participants;

* p - <0.05,

** SNAPPE - Score for Neonatal Acute Physiology Perinatal Extension,

*** DIVA - Difficult Intravenous Access; p value -

1 Kruskal-Wallis,

2 Pearson’schi-square.

The analyses of the primary and secondary outcomes were described in Table 2. The assessment of procedural success demonstrated no statistical significance between the conventional insertion technique and the MST (p=0.705), as well as for the absence of immediate complications (p=0.347). Regarding CLABSI - the only late complication measured, a single case was diagnosed within the 15-day window and the catheter was removed for this reason on the fourth day after insertion. However, the number of attempts for success was equal to three. The variables pain scale, difficulty in hemostasis and procedure time also showed no significance.

**Table 2:** Analysis and comparison of primary outcomes between control and experimental groups, Curitiba, Paraná, Brazil, 2023

Variable	Control Group (n=56)		Experimental Group (n=55)		p^*^
	n (%)	Median (95%CI)	n (%)	Median (95%CI)	
Success					0.705^1^
Yes	26 (48.1)		28 (51.9)		
No	30 (51.7)		28 (48.3)		
Immediate complications					0.347^1^
No	34 (46.6)		39 (53.4)		
Bleeding	13 (52.0)		12 (48.0)		
Hematoma	9 (69.2)		4 (30.8)		
Inadvertent arterial puncture	0 (0.0)		1 (100.0)		
Pain scale		7 (6-12)		7 (5-11)	0.611^2^
Difficulty with hemostasis (dressing up to 48 hours)					0.670^1^
Compression dressing	7 (12.5)		7 (12.7)		
Absence of bleeding	27 (48.2)		30 (54.5)		
Others^**^	22 (39.3)		18 (32.7)		
Procedure time (minutes)		25 (20-30)		27 (20-35)	0.316^2^
Number of attempts		5 (3-6)		4 (3-6)	0.986^2^

n - number of participants;

* p - <0.05;

** Others - catheter fracture/catheter removed soon after insertion due to aberrant trajectory/catheter removed due to obstruction < 24 hours after insertion; p value -

1 Pearson’s chi-square,

2 Kruskal-Wallis.

**Figure 2:**
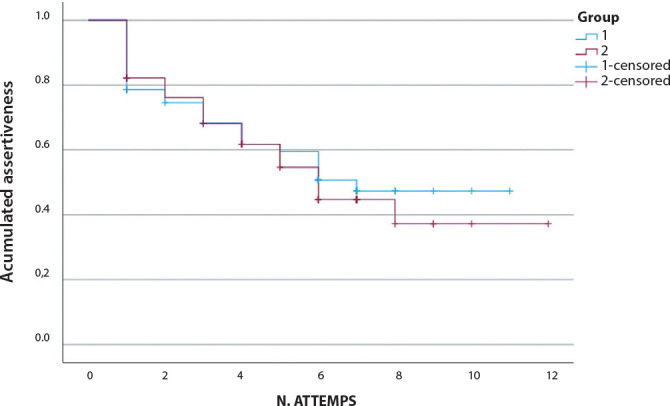
Cumulative percentage of success in relation to the number of attempts between the control and experimental groups, Curitiba, Paraná, Brazil, 2023

Regarding the number of puncture attempts, the CG had 246 versus 242 in the EG. Figure 2 illustrates the cumulative percentage of success in relation to the number of attempts, where the censored cases are those of failure. The Kaplan-Meier curve indicates that all points are very similar, concluding that no insertion technology actually provided a lower number of punctures.

*Group 1* control, Group 2 - experimental, N. - number

The main reasons for catheterization failure were the failure of the PICC (23.4%) or guidewire (3.6%) to progress, psychomotor agitation (5.4%) and inadequate positioning of the tip outside the central vascular system (51.4%). On the other hand, the factors that increased the chances of successful catheterization were the choice of the basilic vein (23.4%), scalp vein (23.4%) and great saphenous vein (18.5%) as target vessels.

Regarding the variables DIVA score, gestational age and birth weight and age on the day of insertion, none of them proved to be relevant for success (Table 3).

**Table 3:** Relationship between clinical and demographic variables and procedural assertiveness, Curitiba, Paraná, Brazil, 2023

Variable	Success (median)	Failure (median)	p^*^
DIVAscore^**^	6 (6-8)	6 (6-7)	0.784
Gestational Age at Birth (weeks)	35 (32-37)	36 (35-38)	0.276
Age at Insertion (days)	33 (17-47)	31 (15-45)	0.725
Birth weight (grams)	2140 (1300-2560)	2470 (2150-3000)	0.136

* p - <0.05,

** DIVA - Difficult Intravenous Access; p value - Kruskal-Wallis.

## DISCUSSION

Currently, most studies have compared the MST and the conventional technology with the use of ultrasound equipment to enhance assertiveness, which is quite well-known. However, only insertion technologies through direct puncture were evaluated in this RCT.

Although our findings do not corroborate the literature in terms of assertiveness and reduction of immediate and late complications, one of the few similar studies, conducted in 2023, demonstrated satisfactory outcomes: the first-attempt success rate was 4.42 times higher in the MST group, the duration of catheterization (p=0.00) and the risk of bleeding after the procedure (p=0.00) were significantly lower compared to the conventional technique^(14)^. Regarding hematoma formation and inadvertent arterial puncture, the microintroduction kit is expected to promote a lower incidence of these events, since the diameter of its introducer is smaller (24 gauge versus 20 gauge). In a venous structure surrounded by poor adiposity, narrow diameter, thin wall, and fewer elastic fibers and smooth muscles, the external force of puncture associated with a large-caliber introducer can easily cause rupture of the blood vessel and unintentional puncture of deeper structures^(14)^. The Infusion Nurses Society^(15)^ recommends puncture with permanent 24-gauge needles to solve these problems.

The main factor related to increased assertiveness in the MST by direct puncture, which is absent in the conventional technique, is the presence of the guidewire in the kit. Immediately after a successful puncture with a 24-gauge needle, the gentle intravascular insertion of the guidewire ensures the maintenance of the catheterized blood vessel, thereby increasing the success of the procedure^(11)^, as the NB’s cooperation cannot be expected and psychomotor agitation is present even when intravenous sedation and analgesia are administered.

Even though there was no difference between the groups in terms of success, another issue to be considered is the learning curve required to incorporate a new technology, which is independent of the previous experience of the professional inserter. A study on the implementation of the MST for PICC insertion in NBs observed that the average time since training for nurses was 13 years and the average experience in neonatology was nine years. The authors concluded that for implementation of the MST, despite proven long experience in the area, professionals needed ongoing and continuing education and assimilated the technology with greater clarity after theoretical and practical training^(16)^. This finding is in line with other studies in which clinical experience is not indicated as a reliable substitute for qualifying professional performance^(17)^. The learning curve of a new technology depends on many factors that vary according to the trainee, the procedure, the instructor, the environment and the level of performance required^(18)^.

Regarding secondary outcomes, the median of the pain scale corresponded to moderate intensity^(19)^. Although this variable did not differ between the groups, when considering the difference in needle gauge between the technologies, the conclusion may be that the thinner one causes less pain, therefore, microintroduction, named precisely for this characteristic, can attenuate the painful stimulus resulting from venipuncture^(11,20)^.

The risk of hemorrhagic complications during insertion after the procedure was monitored in the first two days, since the number of dressing changes within 48 hours is used as an indicator by the Infusion Nurses Society^(15)^. No greater difficulty in hemostasis was observed in the CG, as reported by Wang et al.^(14)^, where 90.2% of insertions in the MST group required only one dressing change within 48 hours and in six cases (9.8%) there were two or more changes within this period. In the CG of the study, there were 38 cases (63.3%) with one dressing change in 48 hours and 21 cases (35%) with two or more changes (p=0.001). Therefore, MST can effectively attenuate bleeding.

Regarding the total procedure time and number of attempts, the comparison between groups was very similar, a result that allows us to conclude that the use of MST was not a factor that reduced attempts or optimized the procedure time. As experts consider the MST a more assertive technique, it consequently helps to reduce the total catheterization time^(14)^. Given the vulnerability of NB added to the numerous painful and invasive stimuli to which they are subjected daily and the risk of exposure to sedation and analgesia, having a procedure that protects them in this context becomes more attractive also in terms of patient safety, neuronal protection, hyperalgesia and allodynia^(21)^.

The number of venipunctures is a variable closely linked to the diagnosis of CLABSI of extraluminal origin. In the first two weeks after insertion, the colonization of the external part of the catheter by microorganisms from the skin predominates^(22)^, and this barrier is broken by the introducer during the puncture(s). The more attempts required the greater the risk of CLABSI and the additional costs, hence the importance of measuring it. Researchers have shown that with each additional venipuncture in NB, the risk of CLABSI and the length of hospital stay increase by 16% and 28 days, respectively, in addition to the cost of €13,850 per episode. Based on these data, they indicated the implementation of the MST to significantly reduce them^(20)^. Likewise, an observational study confirmed this close relationship, even though it used ultrasound: the number of puncture attempts was an independent risk factor for CLABSI^(23)^.

Among the determining reasons for catheterization failure, psychomotor agitation should be discussed. As previously demonstrated, the PIPP score corresponded to moderate pain classification in both groups, even though patients had received mitigating measures for this, findings consistent with the literature^(24,25)^. In this case, two things are relevant. The first is the extreme need for sedation and analgesia in neonatology. The second is the fact that undertreatment of pain interferes with the assertiveness of the procedure, since the NB remains agitated and emotional, making venous cannulation difficult. An analysis performed to identify factors associated with the success of PICC on the first attempt in pediatrics revealed that despite sedation, most children (64.2%) did not cooperate during the procedure. Another notable finding was that even with the help of ultrasound in the MST, assertiveness on the first attempt was low, and achieved only in 59.4% of patients, precisely because psychomotor agitation continued to be present^(11)^.

On the other hand, insertion of the device in the scalp, saphenous and basilic vessels increased the chance of catheterization success. For NB and pediatric patients, in addition to the upper limbs, additional insertion sites are available, including the axillary, temporal and posterior auricular veins, saphenous veins and popliteal veins^(15)^. Considering the basilic vein as the target vessel, the characteristics that make it more assertive are the straight path towards the CAJ, fewer valves that facilitate PICC progression and lower risk of inadvertent arterial puncture^(26)^. Nobre et al.^(27)^ concluded that the basilic vein was more favorable to PICC progression compared to the cephalic vein (p<0.05).

Finally, some sociodemographic and clinical characteristics of the study participants were analyzed in relation to success. Although none of them represented statistical relevance, they are expressive in the literature. A recent study demonstrated that chronicity, low birth weight, and a DIVA score ≥ 4 were independent predictors of difficult peripheral catheterization, and the presence of the latter increased the chances of failure by almost seven times^(28)^. While healthy children undergo, on average, two punctures for successful percutaneous catheterization, DIVA children may experience more than nine attempts, since a score of four or more indicates a 50% increase in the chance of the procedure being unsuccessful^(29,30)^.

The length of stay in the NICU, which in this study was determined by age on the day of insertion, is also related to catheterization failures^(31)^. Newborns undergoing prolonged treatments that require multiple and frequent infusions tend to suffer damage to their peripheral venous network. In the long term, this scenario negatively contributed to the success of PICC^(10,15,32)^.

Regarding age and birth weight, it is widely recognized that PICC placement in NB presents a unique set of technical challenges, which are even more pronounced in premature infants^(8)^. Considering that prematurity is the criterion with the highest score in the DIVA scale, a systematic review concluded that the success rate after the first attempt was significantly lower with decreasing gestational age and associated with greater complications^(33)^.This result was corroborated by several studies^(10,20,34)^.

### Study limitations

There was a delay in starting data collection due to COVID-19 and its health restrictions. Given the characteristics of the intervention by the MST, the blinding of nurses responsible for PICC insertions was not possible. Scientific production on the ultrasound-guided Seldinger technique is predominant over publications that relate it to direct puncture, as was the case of this RCT.

### Contributions to the area of Nursing, health or public policy

There are many contributions from this study, starting with the actual RCT, a necessary step for the production of evidence-based practice that assists in health technology assessments.

Regarding the incorporation of new technologies in the neonatal context, despite having emerged over 70 years ago, the MST has become more widespread and applied in medicine. Therefore, since nurses are the professionals most involved in percutaneous catheterization, they are in an ideal position to conduct prospective studies in this area in order to add robust scientific evidence to the body of knowledge and mitigate the risk of adverse events in NB who require multiple central lines due to device failures before drug treatment is completed.

Little is known about the use of PICC specifically in South American nations. Brazil is one of the largest countries in South America, but little is known about the insertion of percutaneous catheters and their results in the country. Therefore, understanding the use of catheters is very important, as it proves to be a useful marker for the topic in South America.

## CONCLUSIONS

In relation to the successful PICC insertion and the occurrence of immediate and late complications, this clinical research provided evidence that the MST was not a more effective insertion technology compared to the conventional technique.

## Data Availability

*https://doi.org/10.48331/scielodata.WIGV90*
