# Supplementary material for: Effectiveness of the modified Seldinger technique for peripheral central catheter in newborns: a randomized clinical trial
Source: Rev Bras Enferm. 2024 Dec 16;77(6):e20240189. doi: 10.1590/0034-7167-2024-0189 (PMC11654518; doi:10.1590/0034-7167-2024-0189)
Supplement: Translation

# Translation: Efetividade da técnica de Seldinger modificada para cateter central periférico em recém-nascidos: ensaio clínico randomizado

**DOI:** 10.1590/0034-7167-2024-0189pt

**Authors:** Izabela Linha Secco, Mitzy Tannia Reichembach Danski, Luana Lenzi, Higor Pacheco Pereira, Juliana Szreider de Azevedo, Letícia Pontes, Regiane Queiroz Afonso, Camila Fernanda da Silva Milani

**Affiliations:** I Hospital Infantil Waldemar Monastier. Campo Largo, Paraná, Brasil; II Universidade Federal do Paraná. Curitiba, Paraná, Brasil

**Keywords:** Efetividade, Cateterismo Periférico, Recém-Nascido, Tecnologia, Ensaio Clínico Controlado Aleatório.

## Abstract

**Objetivos:**

avaliar a efetividade do cateterismo central periférico entre a técnica de Seldinger modificada e a técnica convencional em recém-nascidos críticos.

**Métodos:**

ensaio clínico randomizado, não mascarado, realizado em um hospital público infantil. Participaram 111 neonatos com alocação randomizada, 56 no grupo controle (tecnologia convencional) e 55 no experimental (microintrodução). O sucesso e a ausência de complicações foram avaliados como desfechos primários. A escala de dor, dificuldade de hemostasia, tempo de procedimento e o número depunções foram considerados desfechos secundários.

**Resultados:**

não houve significância estatística entre os grupos, tanto para o sucesso (p=0,705), como ausência de complicações (p=0,347). No grupo experimental não foi observada menor pontuação da dor, melhora da hemostasia, aumento da assertividade com menos punções e redução do tempo de procedimento.

**Conclusões:**

a microintrodução não se mostrou como uma tecnologia insertora mais efetiva em comparação ao método tradicional. Registro Brasileiro de Ensaio Clínico: RBR-69vks36.

## INTRODUÇÃO

Recém-nascidos (RN) admitidos em Unidades de Terapia Intensiva Neonatal (UTIN) requerem terapia intravenosa prolongada e segura devido à predominância de medicamentos e fluidos que ordenam administração central a longo prazo^(1)^. Sendo assim, a colocação de uma linha central em RN é imprescindível.

Os especialistas têm defendido a transição precoce da terapia de infusão para o Cateter Central de Inserção Periférica (Peripherally Inserted Central Catheter, PICC)^(2)^. As atraentes particularidades do PICC incluem inserção à beira leito, menor taxa de complicações em relação a outros dispositivos centrais, redução de múltiplas punções, desconforto e estresse, além da preservação da rede venosa para uso futuro^(3)^.

Por mais que tenha se tornado um dispositivo factível, seu uso não está insento de complicações e desafios, ambos justificados pela vulnerabilidade dos neonatos, ademais em prematuros. A começar pelo patrimônio venoso, muito mais restrito nessa população comparado às demais faixas etárias^(4)^. Demais atributos que tornam a inserção do PICC tecnicamente exigente em RN são: pele e tecido subcutâneo frouxos, vasos sanguíneos menores e pouco visíveis, falta de cooperação durante o procedimento e anatomia/fisiologia diferenciadas^(5)^.

Tais desafios têm resultado em uma taxa de insucesso no cateterismo de aproximadamente 50% à primeira tentativa^(6)^. O número de tentativas é um fator de risco conhecido para complicações mecânicas, infecciosas e dolorosas. Estas podem ocorrer a cada tentativa adicional durante o cateterismo em pacientes neonatais. Portanto, é relevante ter sucesso em todas as etapas do procedimento na primeira tentativa. Embora a incidência global do cateterismo bem-sucedido seja um desfecho importante, a assertividade na primeira punção é considerada como um parâmetro mais significativo em neonatos^(7)^.

A efetividade da inserção do PICC à primeira tentativa e a redução dos eventos adversos podem ser atingidos a partir de tecnologias e práticas inovadoras, as quais têm evoluído sobremaneira, todas projetadas para melhorar as etapas intraprocedimento^(6)^.

Uma técnica que revolucionou o acesso à rede venosa em pacientes críticos, conhecida como Seldinger, já existe desde a década de 1950, quando um médico radiologista teve a ideia de usar um fio-guia após a punção com agulha para conduzir o cateter dentro do vaso sanguíneo. Com os refinamentos tecnológicos, a técnica evoluiu notavelmente, resultando em menos invasividade na inserção de linhas centrais. Após esses incrementos, a técnica de Seldinger foi modificada (Modified Seldinger Technique - MST) também para atender pacientes que demandam cuidados especiais, como os RN^(8)^.

Em detrimento da técnica convencional, a MST (microintrodução) traz inúmeros benefícios, como a inserção de um calibre maior de cateter, redução da dor, sangramento e do risco de infecção de corrente sanguínea^(9,10)^. E, principalmente, aumento do sucesso na primeira punção, considerando que a progressão do fio-guia no vaso potencializa a chance do PICC ser conduzido até a junção cavo-atrial (JCA)^(11)^. A assertividade ainda pode ser maximizada com o uso do ultrassom, mas a MST independe desse equipamento para ser realizada.

Apesar das duas tecnologias insertoras coexistirem nas UTIN, a convencional ainda é a mais prevalente. O único dispositivo no Brasil que atende aos requisitos da microintrodução para neonatologia foi lançado em 2017 -o Per-Q-Cath^®^NeoKit PICC MST. No tocante à incorporação de novas tecnologias para os neonatos, dispor de uma técnica que proporcione um cateterismo venoso bem-sucedido e reduza as chances de complicações é da maior relevância nos cuidados com essa população.

Diante do exposto, o estudo justifica-se para a prática de enfermagem por incluir uma faixa etária muito frágil e vulnerável, com características peculiares não observadas em nenhuma outra população de pacientes, sobretudo a rede venosa. Outrossim, práticas inovadoras nessa clientela inicialmente perpassam faixas etárias maiores, retardando sua aplicação na prática clínica neonatal.

## OBJETIVOS

Avaliar a efetividade do cateterismo central periférico entre a MST e a técnica convencional em RN criticamente enfermos por meio do sucesso na inserção do PICC e da ocorrência de complicações imediatas e tardias relacionadas a essas tecnologias.

## MÉTODOS

### Aspectos éticos

O estudo foi conduzido de acordo com as diretrizes de ética nacionais e internacionais, sendo aprovado pelo Comitê de Ética em Pesquisa do Setor de Ciências da Saúde - Universidade Federal do Paraná, cujo parecer está anexado à presente submissão. O ensaio clínico foi homologado no Registro Brasileiro de Ensaios Clínicos (RBR-69vks36).

### Desenho, período e local do estudo

Trata-se de um ensaio clínico randomizado (ECR) não mascarado, desenvolvido entre junho de 2022 e novembro de 2023 em um hospital público infantil que é referência no Estado do Paraná. O instrumento norteador do desenho do estudo foi o Consolidated Standards of Reporting Trials (CONSORT).

### População ou amostra, critérios de inclusão e exclusão

O tamanho da amostra foi calculado com base em um estudo do Reino Unido^(12)^, onde a incidência de cateterismo bem-sucedido na primeira tentativa foi de 72% com a MST versus 40% com a técnica convencional. Utilizando um nível de significância (α) de 5%, um poder estatístico de 90% (1-β) e considerando uma possível taxa de abandono de 5%, o tamanho amostral necessário foi de 57 participantes para cada grupo. Contudo, baseado na previsão de que dois kits do cateter experimental estariam vencidos quando as inserções iniciassem, a amostra totalizou 112 pacientes.

Foram incluídos os pacientes admitidos na UTIN a partir do primeiro dia de vida que apresentaram indicação de PICC de acordo com o guideline pediátrico de terapia de infusão miniMAGIC^(13)^. O Consentimento Livre e Esclarecido foi obtido de todos os responsáveis legais dos pacientes por meio escrito. Pacientes com anomalias venosas, infecção de pele, trombocitopênicos (≤ 50.000 mm^3^) e com necessidade de cateterismo venoso central duplo lúmen foram excluídos.

### Protocolo do estudo

O processo de randomização foi realizado por um pesquisador externo experiente em ECR, através de *software* gerado por computador (RANDOM.ORG). Com uso de envelopes opacos e invioláveis contendo a numeração externa de 1 a 112, as alocações de grupo foram ocultadas com as seguintes informações: técnica convencional (GC - Grupo Controle) e MST (GE - Grupo Experimental).

O cegamento dos enfermeiros não foi possível. Antes de iniciarem o procedimento, os mesmos manipulavam o kit do cateter, pois essa etapa faz parte do protocolo pré-inserção. Portanto, sabiam diferenciar as técnicas, visto que o kit MST adiciona alguns itens inexistentes no kit convencional.

A cada indicação de PICC, o paciente era submetido aos critérios de elegibilidade. Sendo elegível, o enfermeiro assistencial fazia contato prévio com a equipe de pesquisa. Esta levava o envelope pardo lacrado até a UTIN, seguindo a randomização previamente realizada, que determinava em que grupo o paciente em questão seria incluído, para identificar posteriormente a tecnologia insertora. A fim de auxiliar nesse controle, foi criado um diário de randomização com o número dos pacientes e o grupo ao qual pertenciam, o qual era preenchido logo após a abertura do envelope pela equipe de pesquisa.

Uma vez alocados aleatoriamente para o uso de uma das tecnologias insertoras, dois enfermeiros assistenciais faziam a avaliação criteriosa da rede venosa e a mensuração estimada do cateter. Na sequência, organizavam os materiais e se paramentavam com barreira máxima estéril. Neste momento, a equipe de pesquisa fazia a abertura do envelope e demonstrava qual kit deveria ser disponibilizado (MST ou convencional). Concomitantemente, a sedoanalgesia era prescrita, preparada e administrada previamente à inserção.

O GE recebeu a intervenção pela técnica MST, enquanto o GC recebeu o tratamento habitual, isto é, a técnica de inserção do PICC já existente no local de estudo. Pela técnica convencional, a intervenção ocorreu através da punção venosa periférica, um centímetro para trás do ponto de inserção desejado. Na presença de refluxo sanguíneo na câmara do introdutor agulhado, a agulha era separada do dilatador peel-away e o cateter era inserido lentamente até o comprimento desejado. Após a progressão total do dispositivo, o peel-away era bipartido até que fosse separado do PICC. O cateter era estabilizado pelas asas de fixação com fita adesiva estéril e uma compressão no sítio de inserção com algodão estéril era aplicada para conter o sangramento. Por fim, a cobertura transparente estéril era colocada sobre o leito de inserção e a localização da ponta do cateter era imediatamente requerida e avaliada por radiografia, antes do início da infusão intravenosa.

A intervenção realizada através da MST compreendeu a punção venosa periférica com um dispositivo agulhado independente do dilatador *peel-away.* Com o retorno venoso, o fio-guia era inserido através da agulha até restar cinco centímetros externamente. Obtendo esta progressão sem dificuldade, a agulha era retirada sobre o fio-guia e este permanecia no vaso do paciente para conduzir a colocação do dilatador peel-away até o final do seu comprimento (sobre o fio-guia). Para a colocação do dilatador, o enfermeiro esticava a pele do neonato para baixo para facilitar a penetração na pele. Então, o fio-guia era retirado e o cateter inserido até o comprimento desejado. As demais etapas foram as mesmas descritas para a técnica convencional.

A equipe de pesquisa, quando avisada pelo enfermeiro assistencial, iniciava o preenchimento do instrumento de coleta de dados através de informações já descritas no prontuário eletrônico, como dados de caracterização dos pacientes e motivos da indicação do PICC. Demais variáveis de interesse eram registradas à beira leito pela equipe, durante e depois do procedimento, abrangendo dados do cateterismo e os desfechos. Obtendo sucesso, o paciente era acompanhado pelos pesquisadores diariamente, até a retirada do cateter.

### Desfechos

As variáveis “sucesso na inserção” e “ausência de complicações imediatas e tardias” foram consideradas como desfechos primários. A primeira abrangeu o posicionamento do PICC em JCA e foi avaliada através da radiografia imediatamente após o fim do procedimento, por um enfermeiro ou médico não participante do estudo.

Em relação às complicações, considerou-se como imediatas a ocorrência de hematoma, sangramento de difícil controle e punção arterial inadvertida, mensuradas durante o cateterismo à beira leito pela equipe de pesquisa. Como complicação tardia, optou-se pela infecção primária de corrente sanguínea, por estar intrinsicamente relacionada ao número de tentativas de punção. Esta foi determinada através de observação diária do paciente, durante as duas primeiras semanas do PICC in situ, através de informações obtidas do prontuário eletrônico e comunicação direta com a equipe de médicos e enfermeiros assistenciais da UTIN.

Os desfechos secundários foram a escala de dor durante o procedimento, a dificuldade de hemostasia no local de inserção até 48 horas, o tempo de procedimento e o número de tentativas de punção, todos também mensurados pelos pesquisadores. Utilizou-se a escala Perfil de Dor do Prematuro (Premature Infant Pain Profile, PIPP) para determinar a presença de dor e graduá-la. O curativo foi visualizado no primeiro e segundo dia após o procedimento, a fim de verificar se precisava ou não de compressão local para conter o sangramento. Nos casos em que o cateterismo foi bem-sucedido, o tempo foi contabilizado em minutos desde a primeira tentativa de punção até a quebra do peel-away. Na ausência de sucesso, o tempo total foi determinado desde a primeira até a última tentativa de punção. O número de tentativas foi definido pela quantidade de vezes em que o neonato foi puncionado.

### Análise dos resultados e estatística

Todas as variáveis de interesse foram transcritas e posteriormente codificadas em uma planilha *Microsoft Excel Office* 365^®^. A análise estatística dos dados ocorreu por meio do *software IBM SPSS Statistics* versão 29.0.

O teste de Kolmogorov-Smirnov foi utilizado para avaliar o padrão de distribuição das variáveis contínuas. Comparações entre variáveis contínuas não paramétricas foram realizadas pelo teste de Kruskal-Wallis, e entre as variáveis categóricas, pelo teste de Qui-quadrado de Pearson. As variáveis categóricas foram descritas utilizando frequência absoluta e relativa; mediana e intervalo de confiança de 95% foram utilizados para descrever as variáveis contínuas. A comparação da assertividade acumulada e o número de tentativas entre os grupos controle e experimental foi realizada pelo método de Kaplan-Meyer.

Em todas as análises considerou-se um nível de significância de 5% (p<0.05), refletindo um intervalo de confiança de 95%.

## RESULTADOS

O recrutamento ocorreu no período de junho de 2022 a novembro de 2023, com a randomização de 56 RN para o GE e 56 para o GC. Durante uma das inserções pela MST, houve a contaminação do cateter e, portanto, perda de um seguimento para o GE (Figura 1).

**Figura 1:** Fluxograma de recrutamento e alocação das participantes do estudo, Curitiba, Paraná, Brasil, 2023

Os dados de base demográficos e as características clínicas de cada grupo estão apresentados na Tabela 1. Os neonatos não diferiram significativamente em termos de idade, peso, risco de mortalidade, diagnóstico médico que culminou na inserção do PICC, escore de acesso venoso difícil e motivos que indicaram a necessidade do cateterismo percutâneo.

**Tabela 1:** Características sociodemográficas e clínicas dos recém-nascidos submetidos ao cateterismo percutâneo, Curitiba, Paraná, Brasil, 2023

Variável	Grupo Controle (n=56)		Grupo Experimental (n=55)		p^*^
	n (%)	Mediana (IC 95%)	n (%)	Mediana (IC 95%)	
Idade gestacional de nascimento (semanas)		36 (34-38)		36 (33-38)	0,785^1^
Idade na inserção (dias)		36 (23-43)		29 (11-59)	0,797^1^
Peso ao nascer (gramas)		2268 (1650-2780)		2355 (1420-2755)	0,969^1^
Escala SNAPPE II^**^		8 (5-15)		5 (5-15)	0,255^1^
Escore DIVA^***^ ≥ 4	34 (48,6)		36 (51.4)		0,605^2^
Indicação do cateter					0,773^2^
Terapia incompatível com via periférica	48 (49,0)		50 (51,0)		
Terapia ≥ 7 dias	10 (47,6)		11 (52,4)		
Acesso venoso difícil	37 (48,7)		39 (51,3)		
Diagnóstico que culminou na inserção do cateter					0,497^2^
Doenças infecciosas e parasitárias	16 (48,5)		17 (51,5)		
Transtornos de outras glândulas endócrinas	1 (100,0)		0 (0,0)		
Doenças do sistema nervoso	1 (33,3)		2 (66,7)		
Doenças do aparelho respiratório	5 (50,0)		5 (50,0)		
Doenças do aparelho digestivo	3 (37,5)		5 (62,5)		
Doenças do aparelho geniturinário	2 (66,7)		1 (33,3)		
Afecções originadas no período neonatal	24 (60,0)		16 (40,0)		
Malformações congênitas, deformidades e anomalias cromossômicas	4 (28,6)		10 (71,4)		

n - número de participantes;

* p - <0,05;

** SNAPPE - Score for Neonatal Acute Physiology Perinatal Extension;

*** DIVA - Difficult Intravenous Access; Valor de p -

1 Kruskal-Wallis,

2 Qui-quadrado Pearson.

As análises dos desfechos primários e secundários foram descritas na Tabela 2. A avaliação do sucesso no procedimento demonstrou que não houve significância estatística entre as técnicas de inserção convencional e MST (p=0,705), assim como para a ausência de complicações imediatas (p=0,347). No que se refere à Infecção Primária de Corrente Sanguínea (IPCS) - única complicação tardia mensurada, um único caso foi diagnosticado dentro da janela de 15 dias: o cateter foi retirado por este motivo no quarto dia após a inserção, porém, o número de tentativas para o sucesso foi igual a três. As variáveis escala de dor, dificuldade de hemostasia e tempo de procedimento também não apresentaram significância.

**Tabela 2:** Análise e comparação dos desfechos primários entre os grupos controle e experimental, Curitiba, Paraná, Brasil, 2023

Variável	Grupo Controle (n=56)		Grupo Experimental (n=55)		p^*^
	n (%)	Mediana (IC 95%)	n (%)	Mediana (IC 95%)	
Sucesso					0,705^1^
Sim	26 (48,1)		28 (51,9)		
Não	30 (51,7)		28 (48,3)		
Complicações imediatas					0,347^1^
Não	34 (46,6)		39 (53,4)		
Sangramento	13 (52,0)		12 (48,0)		
Hematoma	9 (69,2)		4 (30,8)		
Punção arterial inadvertida	0 (0,0)		1 (100,0)		
Escala de dor		7 (6-12)		7 (5-11)	0,611^2^
Dificuldade de hemostasia (curativo até 48 horas)					0,670^1^
Curativo compressivo	7 (12,5)		7 (12,7)		
Ausência de sangramento	27 (48,2)		30 (54,5)		
Outros^**^	22 (39,3)		18 (32,7)		
Tempo procedimento (minutos)		25 (20-30)		27 (20-35)	0,316^2^
Número de tentativas		5 (3-6)		4 (3-6)	0,986^2^

n - número de participantes;

* p - <0,05;

** Outros - cateter fraturou/cateter retirado logo após a inserção por trajeto aberrante/cateter retirado por obstrução < 24 horas de inserção; Valor de p -

1 Qui-quadrado de Pearson,

2 Kruskal-Wallis.

Quanto ao número de tentativas de punção, o GC somou 246 versus 242 no GE. A Figura 2 ilustra o percentual acumulado de sucesso em relação ao número de tentativas, onde os casos censurados são os de insucesso. A curva de Kaplan-Meier indica que todos os pontos são muito parecidos, concluindo que nenhuma tecnologia insertora proporcionou, de fato, menor número de punções.

**Figura 2:** Percentual acumulado de sucesso em relação ao número de tentativas entre os grupos controle e experimental, Curitiba, Paraná, Brasil, 2023

*Grupo 1* controle, Grupo 2 - experimental, N. - número

Os principais motivos determinantes da falha no cateterismo foram a não progressão do PICC (23,4%) ou do fio-guia (3,6%), agitação psicomotora (5,4%) e o posicionamento inadequado da ponta fora do sistema vascular central (51,4%). Por outro lado, os fatores que potencializaram as chances de o cateterismo ser bem-sucedido foram a escolha das veias basílica (23,4%), as do couro cabeludo (23,4%) e a safena magna (18,5%) como vasos alvo.

No tocante às variáveis escore DIVA, idade gestacional e peso de nascimento e idade no dia da inserção, nenhuma delas demonstrou-se relevante para o sucesso (Tabela 3).

**Tabela 3:** Relação entre variáveis clínicas e demográficas com a assertividade no procedimento, Curitiba, Paraná, Brasil, 2023

Variável	Sucesso (mediana)	Insucesso (mediana)	p^*^
Escore DIVA^**^	6 (6-8)	6 (6-7)	0,784
Idade gestacional de nascimento (semanas)	35 (32-37)	36 (35-38)	0,276
Idade na inserção (dias)	33 (17-47)	31 (15-45)	0,725
Peso ao nascer (gramas)	2140 (1300-2560)	2470 (2150-3000)	0,136

* p - <0,05;

** DIVA - Difficult Intravenous Access; Valor de p - Kruskal-Wallis.

## DISCUSSÃO

Atualmente, predominam as pesquisas que compararam a MST com a tecnologia convencional utilizando o equipamento de ultrassom para potencializar a assertividade, a qual é bastante notória. Entretanto, este ECR apenas avaliou tecnologias insertoras por meio de punção direta.

Apesar dos nossos achados não corroborarem com a literatura em termos de assertividade e redução de complicações imediatas e tardias, um dos raros estudos semelhantes, conduzido em 2023, demonstrou desfechos satisfatórios: a taxa de sucesso à primeira tentativa foi 4,42 vezes maior no grupo MST, a duração do cateterismo (p=0,00) e o risco de sangramento após o procedimento (p=0,00) foram significativamente menores em relação à técnica convencional^(14)^. Sobre a formação de hematoma e punção arterial inadvertida, espera-se que o kit de microintrodução promova menor incidência desses eventos, pois o diâmetro de seu introdutor é menor (24 gauge versus 20 gauge). Em uma estrutura venosa circundada de pobre adiposidade, de diâmetro estreito, parede fina, menor quantidade de fibras elásticas e de músculos lisos, a força externa de punção associada a um introdutor calibroso pode facilmente causar a ruptura do vaso sanguíneo e punção não intencional de estruturas mais profundas^(14)^. A *Infusion Nurses Society* ^(15)^ orienta a punção com agulhas permanentes de 24 gauge para solucionar estes problemas.

O principal fator relacionado ao aumento da assertividade na MST por punção direta, inexistente na técnica convencional, é a presença do fio-guia no kit. Logo após uma punção bem-sucedida com uma agulha de 24 gauge, a inserção suave intravascular do guia assegura a manutenção do vaso sanguíneo cateterizado, aumentando, assim, o êxito no procedimento^(11)^, já que não obtemos a colaboração dos RN e a agitação psicomotora se faz presente, ainda que a sedoanalgesia endovenosa seja administrada.

Mesmo não havendo diferença entre os grupos no quesito sucesso, outra questão a ser ponderada diz respeito à curva de aprendizagem necessária à incorporação de uma nova tecnologia, a qual independe da experiência prévia do profissional insertor. Uma pesquisa sobre a implantação da MST para a inserção do PICC em neonatos observou que o tempo médio de formação dos enfermeiros foi de treze anos e a média de experiência em neonatologia de nove anos. Os autores concluíram que para a implantação da MST, embora comprovado muito tempo de experiência na área,os profissionais necessitaram de educação continuada e permanente e assimilaram a tecnologia com maior clareza após a capacitação teórico-prática^(16)^. Essa descoberta está alinhada a outros estudos em que a experiência clínica não é indicada como substituto confiável para qualificar o desempenho profissional^(17)^. A curva de aprendizado de uma nova tecnologia depende de muitos fatores, que variam de acordo com o treinando, o procedimento, o instrutor, o ambiente e o nível de desempenho exigido^(18)^.

Em relação aos desfechos secundários, a mediana da escala de dor correspondeu a uma intensidade moderada^(19)^. Apesar dessa variável não diferir entre os grupos, ao considerar a diferença no calibre das agulhas entre as tecnologias, infere-se que a mais fina cause menos dor, portanto, a microintrodução, nominada justamente por esta característica, pode atenuar o estímulo doloroso decorrente da punção venosa^(11,20)^.

O risco de complicações hemorrágicas na inserção após o procedimento foi acompanhado nos primeiros dois dias, uma vez que o número de trocas de curativo em até 48 horas é utilizado como indicador pela *Infusion Nurses Society* ^(15)^. Não foi observada maior dificuldade de hemostasia no GC, como relataram Wang e colaboradores^(14)^, onde 90,2% das inserções no grupo MST necessitaram de apenas uma troca de curativo em 48 horas e em seis casos (9,8%) houve duas ou mais trocas dentro desse período. Ainda neste estudo, para o GC, ocorreram 38 casos (63,3%) com uma troca de curativo em 48 horas e 21 casos (35%) com duas trocas ou mais (p=0,001). Por isso, a MST pode efetivamente atenuar o sangramento.

No que se refere ao tempo total de procedimento e número de tentativas, a comparação entre os grupos foi muito semelhante, resultado que permite concluir que o uso da MST não foi um fator que reduziu as tentativas nem otimizou o tempo de procedimento. Uma vez a MST sendo considerada por especialistas como uma técnica mais assertiva, consequentemente, colabora para reduzir o tempo total de cateterismo^(14)^. Diante da vulnerabilidade dos RN, somada aos inúmeros estímulos dolorosos e invasivos aos quais são submetidos diariamente e ao risco de exposição à sedoanalgesia, ter um procedimento que os proteja nesse contexto torna-se mais atrativo também em termos de segurança do paciente, proteção neuronal, hiperalgesia e alodinia^(21)^.

O número de punções venosas é uma variável intimamente ligada ao diagnóstico de infecção primária de corrente sanguínea (IPCS) de origem extraluminal. Nas primeiras duas semanas pós-inserção, predomina a colonização da parte externa do cateter por microrganismos da pele^(22)^, barreira esta rompida através do introdutor no ato da(s) punção(ões).Quanto mais tentativas são requeridas, maior o risco de IPCS e os custos adicionais, por isso a importância em mensurá-la. Pesquisadores comprovaram que a cada punção venosa adicional em neonatos, o risco de IPCS e o tempo de internamento aumentam 16% e 28 dias, respectivamente, além do ônus de€13.850 por episódio. Com base nesses dados, indicaram a implementação da MST para reduzi-los significativamente^(20)^. Da mesma forma, um estudo observacional confirmou esta estreita relação, ainda que tenha utilizado a ultrassonografia: o número de tentativas de punção foi um fator de risco independente para a IPCS^(23)^.

Dentro dos motivos determinantes da falha no cateterismo, cabe discutir sobre a agitação psicomotora. Como já demonstrado anteriormente, a pontuação da PIPP correspondeu a uma classificação moderada da dor em ambos os grupos, mesmo os pacientes tendo recebido medidas atenuantes para isso, achados compatíveis com a literatura^(24,25)^. Neste caso, duas coisas são relevantes. A primeira é a extrema necessidade da sedoanalgesia em neonatologia. A segunda é o fato de o subtratamento da dor interferir na assertividade do procedimento, já que o RN permanece agitado e choroso, dificultando a canulação venosa. Uma análise realizada para identificar fatores associados ao sucesso do PICC à primeira tentativa em pediatria revelou que apesar da sedação, a maioria das crianças (64,2%) não cooperou durante o procedimento. Outro achado notável foi que mesmo com o auxílio do ultrassom na MST, a assertividade à primeira tentativa foi baixa, e alcançada somente em 59,4% dos pacientes, justamente pelo fato da agitação psicomotora continuar presente^(11)^.

Por outro lado, a inserção do dispositivo nos vasos do couro cabeludo, safena e basílica aumentaram a chance de sucesso do cateterismo. Para neonatos e pacientes pediátricos, além dos membros superiores, sítios adicionais de inserção estão disponíveis, incluindo as veias axilares, temporais e auriculares posteriores, safenas e poplíteas^(15)^. Considerando a veia basílica como vaso alvo, as características que a tornam mais assertiva são o trajeto retilíneo em direção à JCA, menor número de válvulas, que facilita a progressão do PICC, e menor risco de punção arterial inadvertida^(26)^. Nobre et al.^(27)^ concluíram que a basílica se mostrou mais favorável à progressão do PICC em comparação à veia cefálica (p<0,05).

Por fim, foram analisadas algumas características sociodemográficas e clínicas dos participantes do estudo em relação ao sucesso. Embora nenhuma tenha representado relevância estatística, são expressivas na literatura. Um estudo recente demonstrou que a cronicidade, baixo peso ao nascer e um escore DIVA ≥ 4 foram preditores independentes de cateterismo periférico difícil, sendo que a presença deste último aumentou em quase sete vezes as chances de insucesso^(28)^. Enquanto crianças saudáveis são submetidas, em média, a duas punções para o êxito no cateterismo percutâneo, crianças DIVA podem experimentar mais de nove tentativas, pois uma pontuação a partir de quatro indica um aumento de 50% na chance do procedimento ser mal-sucedido^(29,30)^.

O tempo de permanência em UTIN, neste estudo determinado pela idade no dia da inserção, também está relacionado a falhas no cateterismo^(31)^. Neonatos submetidos a tratamentos prolongados, que exigem infusões múltiplas e frequentes, tendem a sofrer danos em sua rede venosa periférica. A longo prazo, esse cenário contribuiu negativamente para o êxito do PICC^(10,15,32)^.

Em referência à idade e ao peso de nascimento, é amplamente reconhecido que a colocação de PICC em RN apresenta um conjunto único de desafios técnicos, ainda mais potencializados em prematuros^(8)^. Considerando que a prematuridade é o critério de maior pontuação no escore DIVA, uma revisão sistemática concluiu que a taxa de sucesso após a primeira tentativa foi significativamente menor com a diminuição da idade gestacional e associada a maiores complicações^(33)^. Este resultado foi corroborado por várias pesquisas^(10,20,34)^.

### Limitações do estudo

Houve atraso para o início da coleta de dados, em razão da COVID-19 e suas restrições sanitárias. Dadas as características da intervenção pela MST, não foi possível o cegamento dos enfermeiros responsáveis pelas inserções do PICC. A produção científica sobre a técnica de Seldinger guiada por ultrassom é predominante sobre as publicações que a relacionam com a punção direta, como foi o caso deste ECR.

### Contribuições para a área da Enfermagem, saúde ou política pública

Muitas são as contribuições dessa pesquisa. A começar pela condução do ECR propriamente dito, etapa necessária para a produção de prática baseada em evidência, que auxilia nas avaliações de tecnologias em saúde.

No tocante à incorporação de novas tecnologias no contexto neonatal, apesar de ter surgido há mais de 70 anos, a MST tornou-se mais difundida e aplicada na medicina. Por isso, sendo o enfermeiro o profissional mais envolvido no cateterismo percutâneo, sua posição é ideal para realizar estudos prospectivos nessa área, a fim de adicionar evidências científicas robustas ao corpus de conhecimento e mitigar o risco de eventos adversos em neonatos que demandam múltiplas linhas centrais, devido a falhas com dispositivos antes do tratamento medicamentoso concluído.

Especificamente, sabe-se menos sobre o uso do PICC nas nações sul-americanas. O Brasil é um dos maiores países da América do Sul, mas pouco se conhece sobre a inserção de cateteres percutâneos e seus resultados no país. Assim, compreender o seu uso é muito importante, pois se revela como um marcador útil para a temática na América do Sul.

## CONCLUSÕES

Em relação à inserção bem-sucedida do PICC e a ocorrência de complicações imediatas e tardias, esta pesquisa clínica forneceu evidências de que a MST não foi uma tecnologia insertora mais efetiva em comparação à técnica convencional.

## DISPONIBILIDADE DE DADOS E MATERIAL

https://doi.org/10.48331/scielodata.WIGV90
